# Systematic Review of HIV Transmission between Heterosexual Serodiscordant Couples where the HIV-Positive Partner Is Fully Suppressed on Antiretroviral Therapy

**DOI:** 10.1371/journal.pone.0055747

**Published:** 2013-02-13

**Authors:** Mona R. Loutfy, Wei Wu, Michelle Letchumanan, Lise Bondy, Tony Antoniou, Shari Margolese, Yimeng Zhang, Sergio Rueda, Frank McGee, Ryan Peck, Louise Binder, Patricia Allard, Sean B. Rourke, Paula A. Rochon

**Affiliations:** 1 Women’s College Research Institute, Women’s College Hospital, University of Toronto, Toronto, Canada; 2 Faculty of Medicine, University of Toronto, Toronto, Canada; 3 Institute of Health Policy, Management and Evaluation, University of Toronto, Toronto, Canada; 4 St. Michael’s Hospital, University of Toronto, Toronto, Canada; 5 Ontario HIV Treatment Network, Toronto, Ontario, Canada; 6 AIDS Bureau, Ontario Ministry of Health and Long Term Care, Toronto, Canada; 7 HIV and AIDS Legal Clinic Ontario, Toronto, Ontario, Canada; 8 Canadian Treatment Action Council, Toronto, Canada; 9 Canadian HIV/AIDS Legal Network, Toronto, Ontario, Canada; 10 Department of Psychiatry, University of Toronto, Toronto, Ontario, Canada; University of New South Wales, Australia

## Abstract

**Background:**

The risk of sexual HIV transmission in serodiscordant couples when the HIV-positive partner has full virologic suppression on combination antiretroviral therapy (cART) is debated. This study aims to systematically review observational studies and randomized controlled trials (RCTs), evaluating rates of sexual HIV transmission between heterosexual serodiscordant couples when the HIV-positive partner has full suppression on cART.

**Methods and Findings:**

We searched major bibliographic databases to November 2012 for relevant observational studies and RCTs without language restrictions. Conference proceedings, key journals and bibliographies were also searched. Studies reporting HIV transmission rates, cART histories and viral loads of the HIV-positive partners were included. Two reviewers extracted methodologic characteristics and outcomes. Of 20,252 citations, 3 studies met all eligibility criteria with confirmed full virologic suppression in the HIV-positive partner. We included 3 additional studies (2 cohort studies, 1 RCT) that did not confirm viral suppression in the HIV-positive partner at transmission in a secondary meta-analysis. Methodologic quality was reasonable. The rate of transmission in the 3 studies confirming virologic suppression was 0 per 100 person-years (95% CI = 0–0.05), with low heterogeneity (I^2^ = 0%). When we included the 3 studies that did not confirm virologic suppression, the rate of transmission was 0.14 per 100 person-years (95%CI = 0.04–0.31) (I^2^ = 0%). In a sensitivity analysis including all 6 studies, the rate of transmission was 0 per 100 person-years (95%CI = 0–0.01) after omitting all transmissions with known detectable or unconfirmed viral loads, as full suppression in these cases was unlikely. Limitations included lack of data on same-sex couples, type of sexual intercourse (vaginal vs. anal), direction of HIV transmission, exact viral load at the time of transmission, sexually transmitted infections (STI) rates, and extent of condom use.

**Conclusions:**

Our findings suggest minimal risk of sexual HIV transmission for heterosexual serodiscordant couples when the HIV-positive partner has full viral suppression on cART with caveats regarding information on sexual intercourse type, STIs, and condom use. These findings have implications when counseling heterosexual serodiscordant couples on sexual and reproductive health. More research is needed to explore HIV transmission risk between same-sex couples.

## Introduction

The risk of horizontal HIV transmission between serodiscordant couples when the HIV-positive partner has full viral suppression with combination antiretroviral therapy (cART) remains unclear [1]–[3]. This issue is of particular importance to longstanding monogamous discordant couples and to discordant couples considering conception through vaginal heterosexual intercourse [4]–[6]. In the midst of uncertainty regarding the risk of sexual HIV transmission in these contexts, the Swiss National AIDS Commission issued a statement in January 2008 specifying that HIV-infected individuals could be considered non-infectious if they met three conditions: adherence to cART under the care of an HIV physician, virologic suppression below the level of detection for at least six months, and absence of concomitant sexually transmitted infections (STIs) [7]. The “Swiss statement” was highly contentious, particularly in light of evidence demonstrating viral particles in the genital secretions of 5–48% of patients with undetectable plasma viremia [8]–[10].

Since the release of the Swiss Statement [7], 4 systematic reviews have been published quantifying the risk of sexual HIV transmission in serodiscordant couples [11]–[14]. Powers et al (2008) [11] and Boily et al (2009) [12] synthesized evidence on the rates of HIV transmission between HIV-positive individuals and uninfected partners per coital act, with a particular focus on contributing co-factors such as genital ulcer disease, circumcision, and stage of illness. However, neither review accounted for the impacts of antiretroviral use or the viral load of index cases on the risk of transmission. These limitations were somewhat addressed by Attia et al, who conducted a systematic review examining the role of cART use and viral load on HIV transmission between heterosexual discordant couples [13]. However, of the 5 studies included in their review, 3 were conference abstracts and the 2 studies with viral load data were not recent (from 2005 and 2008). The most recent systematic review, available in the Cochrane library examining the impact of cART on sexual transmission of HIV in serodiscordant couples, did not consider the influence of undetectable HIV plasma viral load on the risk of HIV transmission between discordant partners [14]. In addition, these reviews were published prior to the availability of data from the multicenter, randomized controlled trial (RCT), HIV Prevention Trials Network (HPTN) Study 052 [15]. In this trial of 1,763 serodiscordant couples, early initiation of cART was associated with a 96% reduction in the number of linked HIV-1 transmissions relative to delayed cART (i.e. waiting to initiate cART when a clinical event occurred or CD4+ cell count reduced <250 cells/µL) [15].

In summary, existing systematic reviews neither address all factors that could modulate the risk of horizontal HIV transmission between heterosexual serodiscordant couples when the HIV-positive partner has full viral suppression on cART including HIV RNA levels, genital ulcer disease, male circumcision status, and hormonal contraceptive use, nor do they include the most up-to-date evidence surrounding this topic [11], [15]. Consequently, we undertook a systematic review and meta-analysis of quantitative observational studies and RCTs to quantify the risk of horizontal transmission between serodiscordant couples when the HIV-positive partner has full viral suppression on cART. We hypothesize that the risk of horizontal transmission between serodiscordant heterosexual couples when the HIV-positive partner has full viral suppression on cART will be extremely low.

## Methods

### Protocol and Registration

This project was not prospectively registered. A protocol was developed during the planning process (Grant# KRS 102081).

### Literature Search Strategy

This systematic review adheres to guidelines outlined in The Cochrane Group Handbook for Systematic Reviews of Interventions [16]. As well, the PRISMA flow diagram and checklist and MOOSE checklist were used [17], [18]. In consultation with a librarian, a strategy (available in online appendix) was developed for searching MEDLINE (1950– November 2012), EMBASE (1980– November 2012), CINAHL (1980– November 2012), and Web of Science (2004– November 2012) for all relevant observational studies and RCTs regardless of country or language. Keywords searched included “Human immunodeficiency virus”, “HIV transmission”, “intercourse”, and “antiretroviral therapy” (see Appendix for more detail). We also undertook a hand search of journals (online appendix) from June 2010 to November 2012 to identify articles missed by our search, and searched the electronic proceedings of the Conference on Retroviruses and Opportunistic Infections (2008 to 2011), International AIDS Conference (2008 and 2010), and International AIDS Society Conference (2009 and 2011) for relevant abstracts. As only published peer-reviewed full manuscripts were included, authors were contacted by email to ascertain if a full manuscript or publication was available. If any additional information or clarification was required, authors were also contacted.

### Study Selection

Three reviewers divided and screened all citations from the literature search for obvious exclusions. The remaining abstracts were assessed independently by two reviewers (LB, ML), and disagreements were resolved by a third reviewer (MRL). We included published studies of serodiscordant heterosexual or same-sex couples that provided data regarding all of the following: (1) sexual contact, (2) HIV-positive partner taking cART, (3) confirmed undetectable viral load at the time of the HIV transmission, and (4) reported HIV infections rates in HIV-negative partner. The definition of “undetectable viral load” consisted of the viral load being below the level of detection for the year that the test was carried out. This combination of data facilitated our aim to determine the rates of horizontal HIV transmission between serodiscordant couples when the HIV-positive partner has full viral suppression with cART. Studies that did not confirm index case viral loads at the time of transmission were considered for a secondary meta-analysis. As a final step, three experts in the area reviewed our final study selection to identify any missing literature (EM, RP, JM).

### Data Extraction

Two reviewers (MRL, LB) designed and independently piloted a data extraction form. The following items were extracted: study information (location of study, methodology/type of study, study setting, enrolment period, total couples enrolled, total analyzed, follow-up duration, total follow-up in person-years), participants’ demographic information (age, sexual orientation of HIV-positive individual, race/ethnicity), clinical HIV information (type of cART, frequency of HIV testing, frequency of viral load measurement, viral load limit, viral load of index partner, type of assay used), risk factors for HIV transmission (testing of STIs, male circumcision of HIV-positive partner and/or HIV-negative partner, % condom use), and outcome measures reported (HIV transmission on and not on cART, overall HIV transmission rate per 100 person-years and per sexual act, and whether the transmitted virus was genotypically linked). Two investigators (LB, WW) independently assessed each full-text article and extracted the required data. The datasets were compared for each study and a third party settled disagreements (MRL).

### Risk of Bias Assessment

The risk of bias of each eligible study was assessed by two investigators (LB, WW) using the 8-item Newcastle-Ottawa Scale for observational studies [19] (online Appendix) and the Cochrane Risk of Bias tool [20] for RCTs. The datasets were compared and a third party settled disagreements (MRL). These instruments are described in detail elsewhere [19], [20].

### Outcome Measures and Data Synthesis

The primary outcome of interest was incidence of HIV infection in the HIV-negative individual partnered with an HIV-positive person taking cART with fully suppressed viral load. When possible, it was planned to report on incidence of HIV infection per sexual act.

We reported on whether the index case was on cART, whether the HIV transmission occurred on cART, the viral load at the time of transmission, overall HIV transmission rate per 100 person-years, HIV transmission rate per 100 person-years for those on cART and those not on cART, all with 95% confidence intervals (CI[s]). If the exact follow-up time was not available, authors were contacted to attain this information; if there was no reply, it was estimated from the available data. If available, incidence of HIV infection per sexual act was also collected.

### Data Analysis

An a priori decision was made to meta-analyze unadjusted data as adjustment of confounders in observational studies is rare or is inconsistently reported across studies [21]. A meta-analysis was performed of the data reported on the HIV transmission rate per 100 person-years using a fixed-effects Poisson regression model to report the summary HIV transmission rate and 95% CI. Poisson meta-regression was analyzed using a Bayesian framework. Bayesian statistics uses probabilities to make inferences and makes use of new evidence to update prior information [22]. A second meta-analysis was conducted including studies where the HIV-positive partner was on cART with unconfirmed viral suppression at the time of transmission. For this second meta-analysis, the pooled odds ratio (OR) of HIV transmission (cART vs. no cART) was calculated and reported with the 95% CI for eligible studies. Clinical heterogeneity of the studies was assessed and reported in the table of included studies. Statistical heterogeneity was assessed by calculating I^2^ values [23]. A sensitivity analysis using a random-effects model was performed to determine the overall HIV transmission rate per 100 person-years with the 95% CI from only HIV-positive partners on cART with confirmed undetectable viral load. This analysis was carried out by including all studies (those with confirmed and known or unconfirmed suppressed viral loads) but omitting all transmissions with known unsuppressed or unconfirmed viral loads. The meta-analytic software, Comprehensive Meta-analysis Version 2 (Biostat, Englewood NJ [2005]) [24] and Winbugs software release Version 1.4 (Cambridge, UK [2003]) were used [25]. Markov Chains were initiated with three different sets of values and employed 20,000 iterations. The convergence of the model was assessed using the Gelman–Rubin–Brooke statistic [24], [25].

## Results

**Figure 1:**
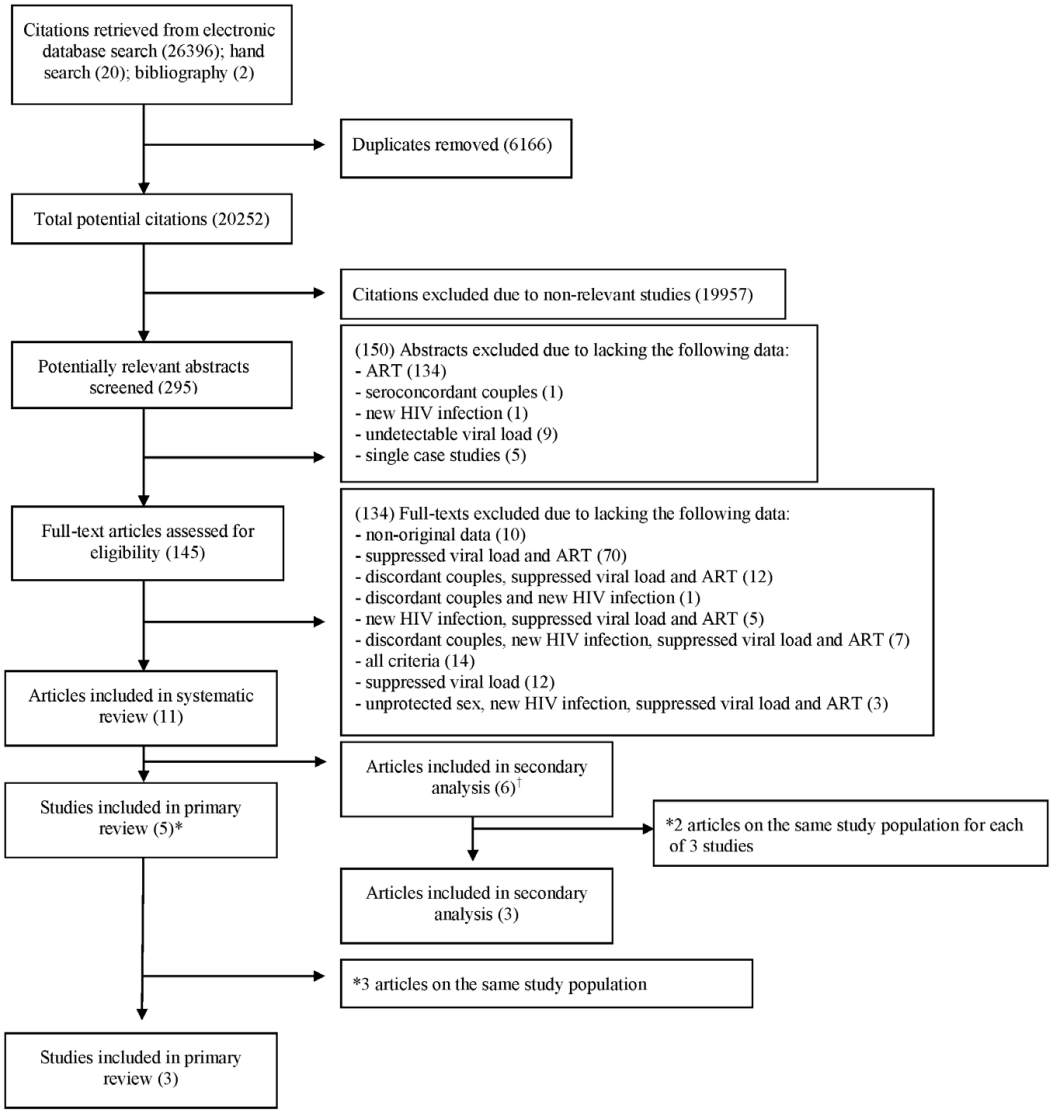
Flow diagram (selection strategy) of included studies.

After removal of duplicate references, 20,252 records from our search of electronic databases were identified, of which 145 were ultimately deemed potentially relevant and retrieved as full-length articles for detailed review (Figure 1). After excluding studies based on design and outcomes of interest, 5 studies from the literature search meeting all inclusion criteria were identified [26]–[30]. Three of the articles reported on the same cohort [28]–[30], leaving 3 studies for analysis. An additional 6 articles met criteria for our secondary meta-analysis and sensitivity analysis as they either had known unsuppressed viral loads or unconfirmed viral load suppression at the time of the transmission [15], [31]–[35]. Three sets of 2 articles reported on the same cohorts, leaving an additional 3 studies for the secondary and sensitivity analyses. The details of the baseline characteristics of the included studies are reported in Table 1. No studies meeting our inclusion criteria provided enough data on same-sex couples, thereby limiting our findings to heterosexual serodiscordant couples. Specifically, there were only 3% same-sex couples in the HPTN 052 study (approximately 53) (gender unspecified). Furthermore, because insufficient data were provided regarding HIV transmission rates per coital act, all subsequent findings are reported by 100 person-years. Also, not enough data were available to present results on transmission rates through intercourse exclusively without condoms, by type of sexual act (vaginal vs. anal), correcting for presence of STIs, or by female-to-male or male-to-female transmission.

**Table 1:** Characteristics of included studies.

Studies	Location	Methodology/type of study	Study setting	Enrolment Period	Age	Gender/sexual orientation of HIV+ partner	Type of cART	Frequency of HIV test	Frequency of viral load measurement	Viral load limit of detection (copies/ml)	Viral load (copies/ml)
**Confirmed Viral Load**											
Melo 2008	Brazil	Retrospective/prospective cohort	Single centre	Feb 2000– Jan 2006	Not reported	Heterosexual 67 (72%) women, 26 (28%) men	Zidovudine, lamivudine, nelfinavir, efavirenz	6 months	Not reported	50	Median: 24082 for transmitter 4583 for non-transmitter All undetectable on ART
Del Romero 2010	Spain	Cross sectional and prospective cohort	Single centre	1989–2008	Median: Women 29 Men 32	Heterosexual 113 (17%) women, 535 (83%) men	Not reported	6 months	Not reported	500 until 1999, 50 thereafter	Median: 6402 for non ART, 5367 for mono/dual therapy, Not detectable for combined treatment
Reynolds 2011	Uganda	Retrospective cohort	Multi mobile clinics	2004–2009	HIV- partner: 5% 15–19 18% 20–24 29% 25–29 48% 30+	Heterosexual 105 (42%) women, 145 (58%) men	Not reported	12 months	6 months	400	6 mo: 71%<400, 29%<2000 12 mo: 85%<400, 15%>2000 24 mo: 100%<400
Studies	Location	Methodology/type of study	Study setting	Enrolment Period	Age	Gender/sexual orientation of HIV+ partner	Type of cART	Frequency of HIV test	Frequency of viral load measurement	Viral load limit of detection (copies/ ml)	Viral load (copies/ml)
**Unconfirmed Viral Load**											
Donnell 2010	Botswana, Kenya, Rwanda, South Africa, Tanzania, Uganda, Zambia	Prospective cohort study	Multi-Centre	Nov 2004– Apr 2007	Median (IQR): HIV+: 32 (26–38) HIV-: 33 (28–40)	Heterosexual 2284 (68%) women, 1097 (32%) men	Stavudine, lamivudine, nevirapine (61%); zidovidine, lamivudine, nevirapine (13%); Protease inhibitor-containing regimen (3%); Other (16%); Insufficient information to establish full regimen (7%)	3 months	Baseline, months 3,6, 12 and final study visit	240	Median: 4.1 log10 copies per ml. 241 (70%) achieved virological suppression at the final visit
Apondi 2011	Uganda	Prospective cohort	Single centre	May 2003– Dec 2007	Median: Women 37 Men 41	Not reported	Not reported	12 months	3 months	50	36 months: 97.5%<1700 2.5%>1700
Cohen 2011	Botswana, Kenya, Malawi, South Africa, Zimbabwe, Brazil, India, Thailand, USA	Randomized controlled trial	Multi-Centre	Jun 2007–May 2010	18%18–25 61% 26–40 21% 40+	97% heterosexual 873 (50%) women, 890 (50%) men	Zidovudine, lamivudine, efavirenz in 72% of participants (Other study drugs: atazanavir, nevirapine, tenofovir, emtricitabine, zidovudine, didanosine, stavudine, lopinavir and ritonavir)	Quarterly	Not reported	400	Median: 4.4 log10 copies per ml

cART, combination antiretroviral therapy; IQR, interquartile range.

The 3 cohort studies with confirmed suppressed virus at the time of HIV transmission reported on 991 heterosexual couples from Brazil, Spain and Uganda with 2,064 person-years of follow up being available [25]–[29]. The additional 2 cohort studies with unconfirmed viral load at the time of HIV transmission were conducted in various countries in Africa and reported on 4,307 couples [31]–[34]. The HPTN 052 RCT was carried out in various African countries, India, Thailand, the United States of America, and Brazil and reported on 1,763 couples. There was 8170 person-years of follow-up for the latter 3 studies [15], [31]–[35].

### Assessment of Risk of Bias and Data from Individual Studies

The results of the risk of bias assessments are reported in Table 2 for the included observational studies and Table 3 for the RCT. Overall, all studies had low risk of bias.

**Table 2:** Risk of bias assessment of included observational studies.

Studies	Representativeness of exposed cohort	Selection of non-exposed cohort	Ascertainment of exposure	Demonstration	Comparability	Assessment of outcome	Follow-up long enough	Adequacy of follow-up	Total score
**Confirmed Viral Load**									
Melo 2008	Somewhat representative*	Same community*	Secure record*	Yes*	No	Medical record*	Yes*	4 of non-ART were lost*	7
Del Romero 2010	Somewhat representative*	Same community*	Structured interview*	Yes*	No	Medical record*	Yes*	65% with follow up	6
Reynolds 2011	Truly representative*	Same community*	Secure record*	Yes*	Study control for behaviour*	Medical record*	Yes*	Not reported	7
**Unconfirmed Viral Load**									
Donnell 2010	Somewhat representative*	Same community*	Secure record*	Yes*	Study control for time on study and CD4 cell count*	Medical record*	Yes*	4% person-years were lost*	8
Apondi 2011	Somewhat representative*	No non-exposed cohort	Secure record*	Yes*	No	Medical record*	Yes*	82% had data at 36 months, 10% died*	6

**Table 3:** Risk of bias assessment of included randomized controlled trial.

Studies	Random sequencegeneration	Allocation concealment	Blinding of participantsand personnel	Blinding of outcome assessment	Incomplete outcome data	Selectivereporting	Other sources of bias
Cohen 2011	Yes	Unclear	Unclear	Unclear	Yes	Yes	Yes

The resulting data from each included study are presented in Table 4. In the 3 cohort studies meeting all inclusion criteria, no transmissions were observed if the HIV-positive partner was taking cART and had undetectable viremia [26]–[30]. In contrast, the 3 studies that had known detectable or unconfirmed viral load data at the time of transmission to the HIV-negative partner recorded 4 such events [15], [31]–[35]. One transmission occurred in each cohort study and 2 in the HPTN 052 study and all were genetically linked. [15], [31]–[35]. Although insufficient detail regarding exact viral load at the time of the transmissions is available, it appears that these events occurred shortly following the initiation of treatment in all cases before full virologic suppression could be attained [15], [31]–[36]. For example, Donnell and colleagues report a female-to-male transmission that occurred within 3 months of the HIV-infected partner initiating cART [31]. Similarly, the genetically linked transmissions from the HPTN 052 study [15], [35] were identified 3 months and 4 weeks after the HIV-positive partner initiated cART, before there was complete viral suppression [15], [30]–[35]. In some of the cases, the viral load was known to be detectable [15], [35], [36].

**Table 4:** Data reported in included studies.

Studies	Total enrolled	Analysed	Follow-up duration	Total follow-up (person-years)	Male circumcision of HIV- partner	Male circumcision of HIV+ partner	Condom use	Index case on cART	HIV transmission on cART	HIV transmission not on cART	HIV transmission rate				
											Per 100 person-years (Overall)	Per 100 person-years (on cART)	Per 100 person-years (Not on cART)	Per 1000 sexual acts (Overall)	Transmitted virus genotypically linked
**Confirmed Viral Load**															
Melo 2008	93	93	Median: 25.5 mo transmitter; 22.34 mo non-transmitter	196.4	No men in the cohort were circumcised	No men in the cohort were circumcised	Interview 37 couples, 8/24 female index case (21.6%) reported no condom use and 13 of 13 men interviewed reported regular condom use	41	0	6	3.1 (1.4–6.5)	0 (0–4.1)	5.7 (2.6–11.8)	Not reported	Not reported
Del Romero 2010	648	648	Not reported	1355	Not reported	Not reported	For patients without ART, 86% had always used condoms	149	0	5	0.4 (0.1–0.9)	0 (0–1.1)	0.6 (0.2–1.4)	0.2 (0.1, 0.6)	Not reported
Reynolds 2011	250	250	Median: 1.57 year before ART, 1.54 year after ART	459.3 before ART, 53.6 after ART	20%	19.3%	Consistent condom use: 14.3% prior to ART, 53.7% after ART	32	0	42	8.2 (6.1–10.9)	0 (0–6.7)	9.2 (6.59, 12.36)	Not reported	Not reported
**Unconfirmed Viral Load**															
Donnell 2010	3408	3381	Median: 8.2 months after ART	4558 for those not on ART, 273 for those on ART	55%	34%	No condom use: 6.2% prior to ART, 3.7% after ART	349	1	102	2.13 (1.76–2.58)	0.37 (0.09–2.04)	2.24 (1.84–2.72)	Not reported	Yes
Apondi 2011	62	62	3 years	Not reported	Not reported	Not reported	Consistent condom use: 74% with discordant partners, 55% with unknown and 46% with concordant partners	62	1	Not applicable	0.5(0.01–3.0)	0.5(0.01–3.0)	Not applicable	Not reported	Yes
Cohen 2011	1763	1775	Median: 1.7 years	1585.3* in early therapy group; 169.5* for delayed-therapy group who started ART; 1397.7* for delayed-therapy group when not on ART	19% of early therapy group and 14% in delayed therapy group	19% of early therapy group and 14% in delayed therapy group	Among HIV infected participants, 96% early-therapy group and 95% delayed-therapy group reported 100% condom use	893	2	27	0.9 (0.6–1.3)	0.1 (0.0–0.4)	2.1 (1.5–3.1)	Not reported	Yes

ART, antiretroviral therapy.

* The follow up duration for those in the early therapy group was 1585.3 person-years as per the Cohen et al publication [15]. Through personal communication [36], we identified that the follow up for the 693 couples in the delayed treatment arm who did not start ART was 1121.2 person-years. There were 184 couples where the HIV-positive started ART, with 276.5 person-years of follow up before the start of ART and 169.5 person-years of follow up after the start of ART.

### Meta-analysis and Heterogeneity Assessment

**Figure 2:**
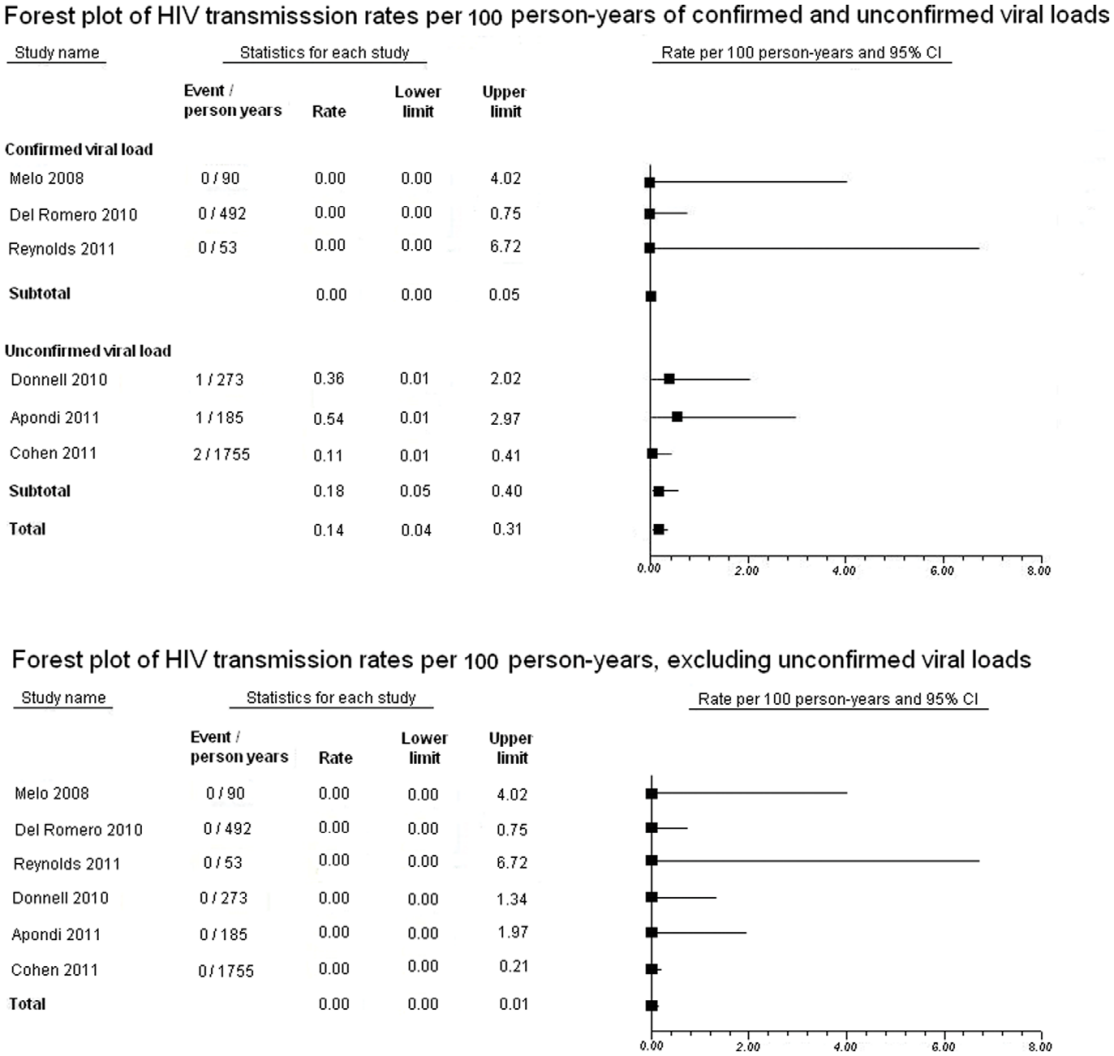
Forest plots of HIV transmission rates per 100 person-years with and without transmissions with unconfirmed viral suppression at the time of suppression. *Footnote:*The first forest plot is the summary of HIV transmission rates per 100 person-years with 95% confidence interval for 6 studies with confirmed and unconfirmed viral suppression at time of transmission. The second forest plot is the sensitivity analysis of the 6 studies with confirmed and unconfirmed viral suppression at the time of transmission with forest plot of the summary of HIV transmission rates per 100 person-years with 95% confidence interval reporting on HIV transmission when HIV-positive partner on combination antiretroviral therapy had confirmed viral suppression, omitting transmissions occurring with known or unconfirmed detectable viral loads at the time of transmission (i.e. 3 studies had 4 transmissions with known or unconfirmed detectable viral loads and these transmissions were excluded, while leaving the rest of data in the analysis).

The first meta-analysis included the 3 cohort studies with confirmed suppressed virus at the time of HIV transmission and is presented in Figure 2 [26]–[30]. The pooled summary HIV transmission rate from these 3 studies was 0 per 100 person-years (95% CI = 0–0.05). The second meta-analysis included all 6 studies, regardless of whether virologic suppression was confirmed at the time of transmission (Figure 2) [15], [26]–[35]. The pooled summary HIV transmission rate from these 6 studies was 0.14per 100 person-years (95% CI = 0.04–0.31). Five of these 6 studies reported on HIV transmission rates in those on and not on cART (all except Apondi et al), allowing a pooled OR to be calculated. For the HPTN 052 study, the authors were contacted and provided the person-year follow up values for the participants in the delayed treatment group who did and did not receive cART [37]. The pooled OR for HIV transmission per 100 person-years for those on versus not on cART from the 5 studies was 0.05(95% CI = 0.01–0.17).

Clinical heterogeneity among the studies is described in Table 1. Statistical heterogeneity was identified for the two meta-analyses and was 0% for both.

### Sensitivity Analysis

As the data suggest that the 4 transmissions for the studies with unconfirmed viral suppression at the time of HIV transmission occurred when the viral load was either known or likely to be unsuppressed [15], [31]–[36], a sensitivity analysis was carried out for the pooled HIV transmission rate per 100 person-years from all HIV-positive partners on cART from the 6 studies excluding these 4 transmissions and is presented in Figure 2. The pooled summary HIV transmission rate from this sensitivity analysis was 0 per 100 person-years (95% CI = 0–0.01).

## Discussion

We performed a comprehensive systematic review of the literature and meta-analyses to assess the risk of HIV sexual transmission between serodiscordant heterosexual partners when the HIV-positive partner has a confirmed undetectable plasma viral load with cART. In our review, only 3 studies where the HIV-positive partner had confirmed full viral suppression on cART were identified [26]–[30], with a pooled transmission rate of 0 per 100 person-years (95% CI = 0–0.05). An additional 3 studies had important data to contribute with a total of 4 transmission events between serodiscordant partners, all occurring early after the start of cART and had known or likely declining viral load but were still detectable [15], [31]–[35]. When included in the meta-analysis, the pooled HIV transmission rate was 0.14 per 100 person-years (95% CI = 0.04–0.31). However, when these transmissions were omitted when viral suppression was unconfirmed in a sensitivity analysis, as detectable virus was known or likely, the pooled HIV transmission rate was 0 per 100 person-years (95% CI = 0–0.01). These findings are useful for the counseling of heterosexual couples considering unprotected intercourse as part of a monogamous consensual relationship [38], [39]. Important caveats exist to our findings including the lack of data on same-sex couples, type of sexual intercourse (vaginal vs. anal), number of sexual encounters in a time period, direction of HIV transmission (male-to-female vs. female-to-male), exact viral load at the time of transmission, STI rates, and extent of condom use. In the counseling setting, it may be difficult to conceptualize the risk per 100 person-years as compared to a person’s lifetime risk of HIV acquisition, which is much more tangible. An HIV transmission rate per 100 person-years can be converted to lifetime risk by multiplying the upper end of our 95% CI (which was 0.0001 in our sensitivity analysis) by 20 to 50 years which is the expected life expectancy of someone diagnosed to be HIV positive who starts on cART [39], [40]. This would translate into approximately a 1 in 204 to 1 in 50 lifetime risk of HIV transmission to the uninfected partner (i.e. 2–5%; which is equivalent to 1% risk per 10 years of relationship and sexual activity).

Our review has several clinical implications. Specifically, our findings emphasize that early initiation of cART is a viable strategy for protection against HIV transmission at the individual level when the viral load is undetectable. Furthermore, our findings can further inform evidence-based discussions between fertility counselors and serodiscordant heterosexual couples [38], [39]. While other groups have performed similar systematic reviews on the topic of HIV sexual transmission between serodiscordant heterosexual partners, our review and meta-analyses underline the importance of viral load in determining sexual HIV transmission in serodiscordant couples. HIV transmission in the studies we analyzed occurred when the participants were not taking cART, had detectable plasma HIV-1 viral load, or were in the window period between initiating cART and having a confirmed suppressed viral load. Powers et al (2008) [11] and Boily et al (2009) [12] did not report critical factors such as viral load and the influence of cART. Attia et al (2009) [13] integrated published and unpublished literature in their investigation. Based on two studies, they reported zero episodes of seroconversion from cART-treated sero-positive partners whose viral loads were <400 copies/ml and reported one transmission per 79 person-years as the upper limit of the 95% CI with 291 person-years of follow-up [13]. In their review, a 92% reduction rate of HIV transmission was established when comparing cART-exposed index cases relative to those not on cART [13]. However, their rates included studies only presented in conference abstract form and from studies not updated past 2008 [13]. Anglemeyer et al (2011) reported 71 out of 436 episodes of transmission amongst couples treated with cART, but did not indicate index case viral loads to be able to comment on the role of suppressed viral load during these transmissions [14]. In a similar fashion to how Attia et al reported, the upper limit of the 95% CI for our sensitivity analysis revealed one transmission in 1,000 person-years from data with 2,975 person-years of follow-up, more person-years being reported on the topic to date.

Our systematic review’s findings do not contradict the Swiss National AIDS Commission statement that HIV-positive individuals are sexually non-infectious if they are fully virologically suppressed for at least 6 months with consistent adherence to cART and if no STIs are present [7]. Our review found that the rate of HIV-1 transmission for cART-treated patients with documented suppressed plasma viral load was 0 per 100-person years. However, our data only applies to heterosexual couples with some degree of variable condom use and does not incorporate impact of STIs. As published studies in same-sex couples were done using modeling and not with prospective data collection, our results must be used with caution and can only be inferred for this population.

Again, several limitations of our systematic review merit emphasis. Most notably, the impact of consistent condom use on the rate of HIV transmission could not be accounted for by most of the studies included in our review. In addition, most studies did not provide data regarding the presence of concomitant STIs, the prevalence of hormonal contraceptive use, and the risk of HIV transmission per coital act. Furthermore, extrapolation of these findings to same-sex couples is limited. The studies which have been conducted to date are of limited treatment length of time and do not account for durability of viral suppression. Also, type of sexual intercourse (vaginal vs. anal), number of sexual encounters in a time period, and direction of HIV transmission (male-to-female vs. female-to-male) were not reported. These limitations must be considered when applying these findings to the clinical setting, and highlight additional areas where further research is required.

Again, a notable limitation of our review is the lack of data provided on details of exact condom use. This has made it impossible to calculate risk through intercourse exclusively without condoms. In the HPTN 052 study, 5% and 6% of the HIV-positive and HIV-negative participants, respectively, reported unprotected intercourse in the week prior to enrolment [15]. This variable of self-reported condom use <100% at baseline was associated with significant increased transmission [15], however details are not provided.

Other important limitation of our study include that 1) the exact viral load of the HIV-positive partner at the time of transmission is difficult to capture as it is impractical to carry out daily viral load testing. Therefore, our selection criteria ’confirmed undetectable viral load at the time of the HIV transmission” poses some methodological difficulty as the number of studies with the known viral load of the HIV-positive partner at the time of transmission is low. This has led us to a bias of including studies where there was no HIV transmission in our primary analysis. Viral suppression and time of infection are time-varying variables that can only ever be measured at discrete time and rather should be thought of as observed viral suppression at the beginning and end of a time interval around the occurrence of a new infection; 2) the studies included in our systematic review did not include data on genital viral load. However, it can be deduced based on previous evidence that 5–48% of the HIV-positive patients with fully suppressed viral loads have detectable HIV-1 RNA in their genital secretion [8]–[10]. It has been the subject of controversy whether the presence of such genital HIV-1 RNA possesses any clinical significant risk to transmission. From our review, it appears to have had no influence on the results and did not lead to HIV transmission in the studies under review; and 3) there was a lack of data on STI rates and the impact of the presence of STIs. In summary, the limitations listed above culminate in the weakness that our study was unadjusted, which is understandably a difficult procedure in such analyses.

### Conclusions

In summary, our systematic review contributes to the emerging body of literature expanding on the position put forth by the Swiss National AIDS Commission [7] that unprotected sexual intercourse is a viable conception and sexual option for heterosexual serodiscordant couples in monogamous relationships if the HIV-infected partner has full virologic suppression on cART, where both parties understand the limitations of the available data. Our research is important given the increasing importance of parenthood and healthy sexuality on the overall well-being of the lives of HIV-positive individuals and their partners [41]–[45]. Additional research is required to clarify the impact of cART on HIV transmission in serodiscordant same-sex couples and the impact of STIs, type of sexual intercourse and actual transmission rate with confirmed unprotected intercourse.
