# Correction: Systematic Review of HIV Transmission between Heterosexual Serodiscordant Couples where the HIV-Positive Partner Is Fully Suppressed on Antiretroviral Therapy

**DOI:** 10.1371/annotation/aa7792b2-f841-4b23-a808-384ba1b35ae4

**Published:** 2013-12-09

**Authors:** Mona R. Loutfy, Wei Wu, Michelle Letchumanan, Lise Bondy, Tony Antoniou, Shari Margolese, Yimeng Zhang, Sergio Rueda, Frank McGee, Ryan Peck, Louise Binder, Patricia Allard, Sean B. Rourke, Paula A. Rochon

There are errors in the last two sentences of the first paragraph of the "Discussion" section. The correct sentences are:

An HIV transmission rate per person-years can be converted to lifetime risk by multiplying the upper end of our 95% CI (which was 0.0001 in our sensitivity analysis) by 20 to 50 years which is the expected life expectancy of someone diagnosed to be HIV positive who starts on cART [39,40]. This would translate into approximately a 1 in 200 to 1 in 500 lifetime risk of HIV transmission to the uninfected partner (i.e. 0.2–0.5%; which is equivalent to 0.1% risk per 10 years of relationship and sexual activity).

Additionally, there is an error in the last sentence of the second paragraph of the "Discussion" section. The correct sentence is:

In a similar fashion to how Attia et al reported, the upper limit of the 95% CI for our sensitivity analysis revealed one transmission in 10,000 person-years from data with 2,848 person-years of follow-up, more person-years being reported on the topic to date. 

